# Bis{μ-2-[2-(2-pyrid­yl)ethyl­imino­meth­yl]phenolato}bis­[azido­zinc(II)]

**DOI:** 10.1107/S1600536808013184

**Published:** 2008-05-10

**Authors:** Xiang-Rong Zhou, Zhong-Shu Li, Lei Zhang, Zhi-Hong Zhou

**Affiliations:** a Ordered Matter Science Research Center, College of Chemistry and Chemical Engineering, Southeast UniVersity, Nanjing 210096, People’s Republic of China

## Abstract

In the centrosymmetric title dinuclear zinc(II) compound, [Zn_2_(C_14_H_13_N_2_O)_2_(N_3_)_2_], each Zn^II^ ion has a slightly distorted trigonal bipyramidal geometry and is coordinated by two N atoms and one O atom from one Schiff base ligand, an O atom from the other Schiff base ligand, and another N atom from an azide ligand. The crystal structure involves inter­molecular C—H⋯N hydrogen bonds.

## Related literature

For related literature, see: Tandon *et al*. (2000 ▶); Fu & Ye (2007 ▶); Li & Zhang (2004 ▶); You & Zhu (2004 ▶).
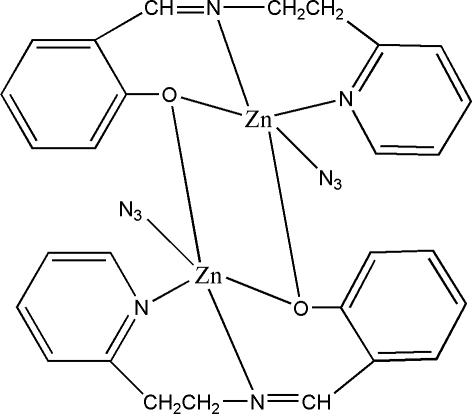


## Experimental

### 

#### Crystal data

[Zn_2_(C_14_H_13_N_2_O)_2_(N_3_)_2_]
*M* *_r_* = 665.37
Monoclinic, 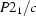

*a* = 9.523 (9) Å
*b* = 9.466 (9) Å
*c* = 15.853 (14) Å
β = 100.664 (17)°
*V* = 1404 (2) Å^3^
*Z* = 2
Mo *K*α radiation
μ = 1.75 mm^−1^
*T* = 293 (2) K
0.05 × 0.05 × 0.05 mm

#### Data collection

Bruker SMART 1K CCD area-detector diffractometer
Absorption correction: multi-scan (*CrystalClear*; Rigaku, 2005 ▶) *T*_min_ = 0.915, *T*_max_ = 0.915
11424 measured reflections
2460 independent reflections
1824 reflections with *I* > 2σ(*I*)
*R*_int_ = 0.117

#### Refinement

*R*[*F* ^2^ > 2σ(*F* ^2^)] = 0.062
*wR*(*F* ^2^) = 0.154
*S* = 1.00
2460 reflections
190 parameters
H-atom parameters constrained
Δρ_max_ = 0.32 e Å^−3^
Δρ_min_ = −0.37 e Å^−3^

### 

Data collection: *CrystalClear* (Rigaku, 2005 ▶); cell refinement: *CrystalClear*; data reduction: *CrystalClear*; program(s) used to solve structure: *SHELXS97* (Sheldrick, 2008 ▶); program(s) used to refine structure: *SHELXL97* (Sheldrick, 2008 ▶); molecular graphics: *SHELXTL* (Sheldrick, 2008 ▶); software used to prepare material for publication: *SHELXTL*.

## Supplementary Material

Crystal structure: contains datablocks I, global. DOI: 10.1107/S1600536808013184/bq2072sup1.cif

Structure factors: contains datablocks I. DOI: 10.1107/S1600536808013184/bq2072Isup2.hkl

Additional supplementary materials:  crystallographic information; 3D view; checkCIF report

## Figures and Tables

**Table 1:** Hydrogen-bond geometry (Å, °)

*D*—H⋯*A*	*D*—H	H⋯*A*	*D*⋯*A*	*D*—H⋯*A*
C9—H9*A*⋯N5^i^	0.97	2.58	3.198 (9)	122

Symmetry code: (i) 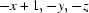.

**Fig. 1.**
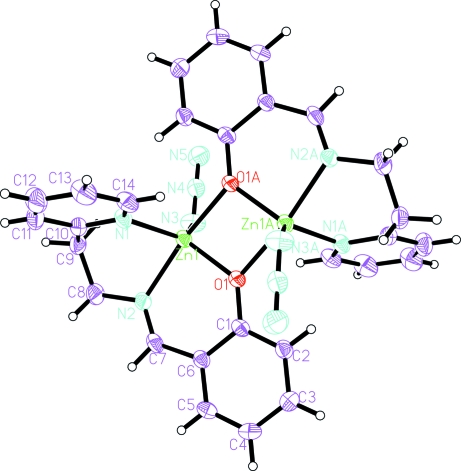
The molecular structure of the title compound with the atom-numbering scheme. Displacement ellipsoids are drawn at the 30% probability level. [Symmetry code i : -x, -y, -z ]

**Fig. 2.**
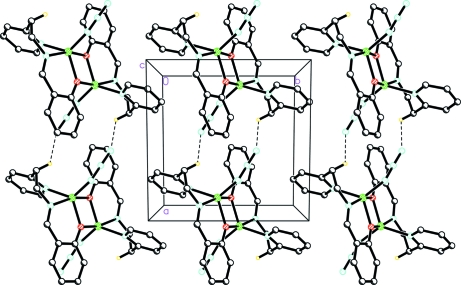
One-dimensional structure in the title compound. Hydrogen bonds are shown as dashed lines. Displacement ellipsoids are drawn at the 30% probability level and all hydrogen atoms, except H9a was omitted for clarity.

## supplementary crystallographic information

### Comment

Transition metal compounds containing Schiff base ligands are of great interest since many years. These compounds play an important role in the development of coordination chemistry related to their potential applications in catalysis and enzymatic reactions, magnetism and molecular architecture (You & Zhu, 2004; Li & Zhang, 2004).

We have focused on the synthesis of Schiff base complex which is formed by Cu(CH_3_COO)_2_.H_2_O, schiff base ligand 2-(pyridin-2-ylethyliminomethyl)phenol and sodium azide. The title dinuclear zinc(II) complex is reported here.

As shown in Fig. 1, the molecule of the title compound is composed of two Zn^II^ atoms, two schiff base ligand 2-(pyridin-2-ylethyliminomethyl)phenol and two azido. Each Zn^II^ atom shows a slightly distorted trigonal-bipyramidal geometry (Fu & Ye, 2007) formed by two N atoms and one O atom from one schiff base ligand (S.S. Tandon *et al.*, 2000), the another O atom of the second schiff base, together with another N atom from azido. There are intermolecular C—H···N hydrogen bonds in the crystal structure leading to a one-dimensional supramolecular structure (Fig. 2 and Table 1).

### Experimental

The title compound was synthesized by Cu(CH_3_COO)_2_.H_2_O, schiff base ligand 2-(pyridin-2-ylethyliminomethyl)phenol and sodium azide. All chemicals used (reagent grade) were commercially available. Salicylaldehyde (0.122 g, 1 mmol) was dissolved in ethanol (5 mL) and ethanol solution (5 mL) containing 2-aminoethylpyridine (0.108 g, 1 mmol) was added slowly with stirring. The resulting yellow solution was continuously stirred for about 30 min. at room temperature, and then Cu(CH_3_COO)_2_.H_2_O (0.200 g, 1 mmol) and sodium azide (0.13 g 2 mmol) in aqueous solution (5 mL) was added with stirring homogeneously. Colorless crystals suitable for X-ray analysis were obtained by slow evaporation at room temperature over several days.

### Refinement

Positional parameters of all H atoms were calculated geometrically.

### Crystal data

**Table:**

[Zn_2_(C_14_H_13_N_2_O_1_)_2_(N_3_)_2_]	*F*_000_ = 680
*M**_r_* = 665.37	*D*_x_ = 1.575 Mg m^−^^3^
Monoclinic, *P*2_1_/*c*	Mo *K*α radiation λ = 0.71073 Å
Hall symbol: -P 2ybc	Cell parameters from 2415 reflections
*a* = 9.523 (9) Å	θ = 3.1–27.5º
*b* = 9.466 (9) Å	µ = 1.75 mm^−^^1^
*c* = 15.853 (14) Å	*T* = 293 (2) K
β = 100.664 (17)º	Block, colorless
*V* = 1404 (2) Å^3^	0.05 × 0.05 × 0.05 mm
*Z* = 2	

### Data collection

**Table:**

Bruker SMART 1K CCD area-detector diffractometer	2460 independent reflections
Radiation source: fine-focus sealed tube	1824 reflections with *I* > 2σ(*I*)
Monochromator: graphite	*R*_int_ = 0.117
Detector resolution: 8.192 pixels mm^-1^	θ_max_ = 25.0º
*T* = 293(2) K	θ_min_ = 3.1º
thin–slice ω scans	*h* = −11→11
Absorption correction: Multi-scan(CrystalClear; Rigaku, 2005)	*k* = −11→11
*T*_min_ = 0.915, *T*_max_ = 0.915	*l* = −18→18
11424 measured reflections	

### Refinement

**Table:**

Refinement on *F*^2^	Secondary atom site location: difference Fourier map
Least-squares matrix: full	Hydrogen site location: inferred from neighbouring sites
*R*[*F*^2^ > 2σ(*F*^2^)] = 0.062	H-atom parameters constrained
*wR*(*F*^2^) = 0.154	*w* = 1/[σ^2^(*F*_o_^2^) + (0.0733*P*)^2^] where *P* = (*F*_o_^2^ + 2*F*_c_^2^)/3
*S* = 1.00	(Δ/σ)_max_ < 0.001
2460 reflections	Δρ_max_ = 0.32 e Å^−^^3^
190 parameters	Δρ_min_ = −0.37 e Å^−^^3^
Primary atom site location: structure-invariant direct methods	Extinction correction: none

### Special details

**Table:**

Geometry. All e.s.d.'s (except the e.s.d. in the dihedral angle between two l.s. planes) are estimated using the full covariance matrix. The cell e.s.d.'s are taken into account individually in the estimation of e.s.d.'s in distances, angles and torsion angles; correlations between e.s.d.'s in cell parameters are only used when they are defined by crystal symmetry. An approximate (isotropic) treatment of cell e.s.d.'s is used for estimating e.s.d.'s involving l.s. planes.
Refinement. Refinement of *F*^2^ against ALL reflections. The weighted *R*-factor *wR* and goodness of fit *S* are based on *F*^2^, conventional *R*-factors *R* are based on *F*, with *F* set to zero for negative *F*^2^. The threshold expression of *F*^2^ > σ(*F*^2^) is used only for calculating *R*-factors(gt) *etc*. and is not relevant to the choice of reflections for refinement. *R*-factors based on *F*^2^ are statistically about twice as large as those based on *F*, and *R*- factors based on ALL data will be even larger.

### Fractional atomic coordinates and isotropic or equivalent isotropic displacement parameters (Å^2^)

**Table:**

	*x*	*y*	*z*	*U*_iso_*/*U*_eq_	
Zn1	0.89278 (7)	0.07610 (6)	0.05279 (4)	0.0388 (3)	
O1	1.0963 (4)	0.0175 (4)	0.0700 (2)	0.0443 (10)	
N2	0.9352 (5)	0.1930 (5)	0.1691 (3)	0.0437 (12)	
C2	1.3382 (6)	−0.0122 (6)	0.1341 (4)	0.0439 (14)	
H2A	1.3492	−0.0743	0.0904	0.053*	
N1	0.8313 (5)	0.2740 (5)	−0.0024 (3)	0.0437 (12)	
C1	1.2057 (6)	0.0467 (5)	0.1339 (3)	0.0387 (13)	
C7	1.0588 (7)	0.2101 (6)	0.2129 (4)	0.0460 (14)	
H7A	1.0666	0.2750	0.2576	0.055*	
C6	1.1919 (6)	0.1403 (6)	0.2022 (3)	0.0407 (14)	
C5	1.3113 (7)	0.1695 (6)	0.2653 (4)	0.0513 (16)	
H5A	1.3017	0.2302	0.3099	0.062*	
N4	0.6662 (7)	−0.1265 (5)	0.0553 (3)	0.0491 (13)	
C10	0.7506 (6)	0.3590 (6)	0.0385 (4)	0.0448 (14)	
N3	0.7675 (7)	−0.0641 (6)	0.0914 (4)	0.0615 (15)	
C8	0.8169 (7)	0.2774 (7)	0.1912 (4)	0.0578 (17)	
H8A	0.8531	0.3685	0.2133	0.069*	
H8B	0.7782	0.2296	0.2360	0.069*	
N5	0.5662 (7)	−0.1902 (6)	0.0247 (4)	0.0684 (16)	
C3	1.4564 (7)	0.0186 (7)	0.1979 (4)	0.0528 (16)	
H3B	1.5443	−0.0231	0.1964	0.063*	
C11	0.7189 (8)	0.4943 (7)	0.0084 (5)	0.0626 (19)	
H11A	0.6620	0.5516	0.0358	0.075*	
C14	0.8829 (7)	0.3237 (6)	−0.0705 (4)	0.0536 (16)	
H14A	0.9394	0.2653	−0.0975	0.064*	
C9	0.6990 (7)	0.2995 (7)	0.1140 (4)	0.0527 (16)	
H9A	0.6526	0.2096	0.0980	0.063*	
H9B	0.6280	0.3628	0.1298	0.063*	
C4	1.4422 (8)	0.1111 (7)	0.2632 (4)	0.0618 (18)	
H4A	1.5207	0.1335	0.3053	0.074*	
C13	0.8535 (9)	0.4595 (7)	−0.1009 (5)	0.071 (2)	
H13A	0.8901	0.4920	−0.1477	0.085*	
C12	0.7695 (9)	0.5460 (7)	−0.0612 (5)	0.076 (2)	
H12A	0.7476	0.6373	−0.0810	0.091*	

### Atomic displacement parameters (Å^2^)

**Table:**

	*U*^11^	*U*^22^	*U*^33^	*U*^12^	*U*^13^	*U*^23^
Zn1	0.0443 (4)	0.0393 (4)	0.0344 (4)	0.0026 (3)	0.0112 (3)	−0.0035 (3)
O1	0.041 (2)	0.051 (2)	0.040 (2)	0.0056 (19)	0.0049 (19)	−0.0138 (18)
N2	0.052 (3)	0.047 (3)	0.033 (3)	0.009 (2)	0.010 (2)	−0.001 (2)
C2	0.044 (4)	0.045 (3)	0.045 (4)	−0.001 (3)	0.013 (3)	−0.007 (3)
N1	0.056 (3)	0.039 (3)	0.037 (3)	0.002 (2)	0.013 (2)	−0.001 (2)
C1	0.054 (4)	0.037 (3)	0.027 (3)	−0.004 (3)	0.011 (3)	0.002 (2)
C7	0.066 (4)	0.040 (3)	0.035 (3)	0.004 (3)	0.016 (3)	−0.006 (2)
C6	0.053 (4)	0.035 (3)	0.034 (3)	0.002 (3)	0.008 (3)	0.000 (2)
C5	0.059 (4)	0.056 (4)	0.036 (4)	−0.002 (3)	0.003 (3)	−0.012 (3)
N4	0.066 (4)	0.043 (3)	0.041 (3)	0.007 (3)	0.017 (3)	0.008 (2)
C10	0.049 (4)	0.037 (3)	0.046 (4)	0.006 (3)	0.004 (3)	−0.002 (3)
N3	0.067 (4)	0.072 (4)	0.045 (3)	−0.022 (3)	0.009 (3)	0.009 (3)
C8	0.060 (4)	0.067 (4)	0.051 (4)	0.013 (3)	0.022 (3)	−0.009 (3)
N5	0.066 (4)	0.060 (4)	0.076 (5)	−0.003 (3)	0.004 (3)	0.005 (3)
C3	0.044 (4)	0.056 (4)	0.057 (4)	0.003 (3)	0.007 (3)	−0.002 (3)
C11	0.077 (5)	0.047 (4)	0.065 (5)	0.017 (4)	0.016 (4)	0.003 (3)
C14	0.069 (4)	0.052 (4)	0.041 (4)	−0.008 (3)	0.014 (3)	−0.005 (3)
C9	0.053 (4)	0.053 (4)	0.055 (4)	0.013 (3)	0.017 (3)	−0.003 (3)
C4	0.054 (4)	0.072 (5)	0.052 (4)	−0.004 (4)	−0.009 (3)	−0.011 (3)
C13	0.107 (6)	0.048 (4)	0.058 (5)	−0.016 (4)	0.016 (4)	0.007 (3)
C12	0.115 (7)	0.038 (4)	0.070 (6)	0.003 (4)	0.005 (5)	0.005 (3)

### Geometric parameters (Å, °)

**Table:**

Zn1—N3	1.957 (6)	N4—N5	1.155 (7)
Zn1—O1	1.986 (4)	N4—N3	1.185 (7)
Zn1—N1	2.104 (5)	C10—C11	1.380 (8)
Zn1—N2	2.125 (5)	C10—C9	1.487 (8)
Zn1—O1^i^	2.158 (4)	C8—C9	1.515 (8)
O1—C1	1.342 (6)	C8—H8A	0.9700
O1—Zn1^i^	2.158 (4)	C8—H8B	0.9700
N2—C7	1.262 (7)	C3—C4	1.381 (9)
N2—C8	1.475 (7)	C3—H3B	0.9300
C2—C1	1.378 (8)	C11—C12	1.373 (10)
C2—C3	1.398 (8)	C11—H11A	0.9300
C2—H2A	0.9300	C14—C13	1.383 (9)
N1—C14	1.350 (7)	C14—H14A	0.9300
N1—C10	1.358 (7)	C9—H9A	0.9700
C1—C6	1.423 (7)	C9—H9B	0.9700
C7—C6	1.467 (8)	C4—H4A	0.9300
C7—H7A	0.9300	C13—C12	1.375 (10)
C6—C5	1.396 (8)	C13—H13A	0.9300
C5—C4	1.369 (9)	C12—H12A	0.9300
C5—H5A	0.9300		
			
N3—Zn1—O1	113.8 (2)	N1—C10—C11	119.5 (6)
N3—Zn1—N1	126.5 (2)	N1—C10—C9	117.4 (5)
O1—Zn1—N1	119.68 (18)	C11—C10—C9	123.1 (6)
N3—Zn1—N2	96.3 (2)	N4—N3—Zn1	132.5 (5)
O1—Zn1—N2	90.16 (17)	N2—C8—C9	111.6 (5)
N1—Zn1—N2	83.74 (18)	N2—C8—H8A	109.3
N3—Zn1—O1^i^	97.9 (2)	C9—C8—H8A	109.3
O1—Zn1—O1^i^	78.48 (16)	N2—C8—H8B	109.3
N1—Zn1—O1^i^	92.66 (17)	C9—C8—H8B	109.3
N2—Zn1—O1^i^	164.47 (18)	H8A—C8—H8B	108.0
C1—O1—Zn1	130.4 (3)	C4—C3—C2	119.8 (6)
C1—O1—Zn1^i^	127.3 (3)	C4—C3—H3B	120.1
Zn1—O1—Zn1^i^	101.52 (16)	C2—C3—H3B	120.1
C7—N2—C8	118.4 (5)	C10—C11—C12	121.4 (6)
C7—N2—Zn1	123.5 (4)	C10—C11—H11A	119.3
C8—N2—Zn1	117.2 (4)	C12—C11—H11A	119.3
C1—C2—C3	122.1 (5)	N1—C14—C13	121.5 (6)
C1—C2—H2A	118.9	N1—C14—H14A	119.3
C3—C2—H2A	118.9	C13—C14—H14A	119.3
C14—N1—C10	119.8 (5)	C10—C9—C8	113.4 (5)
C14—N1—Zn1	121.9 (4)	C10—C9—H9A	108.9
C10—N1—Zn1	117.9 (4)	C8—C9—H9A	108.9
O1—C1—C2	120.0 (5)	C10—C9—H9B	108.9
O1—C1—C6	122.4 (5)	C8—C9—H9B	108.9
C2—C1—C6	117.6 (5)	H9A—C9—H9B	107.7
N2—C7—C6	128.1 (5)	C5—C4—C3	119.3 (6)
N2—C7—H7A	116.0	C5—C4—H4A	120.3
C6—C7—H7A	116.0	C3—C4—H4A	120.3
C5—C6—C1	119.4 (5)	C12—C13—C14	119.4 (7)
C5—C6—C7	115.7 (5)	C12—C13—H13A	120.3
C1—C6—C7	124.9 (5)	C14—C13—H13A	120.3
C4—C5—C6	121.8 (6)	C13—C12—C11	118.4 (6)
C4—C5—H5A	119.1	C13—C12—H12A	120.8
C6—C5—H5A	119.1	C11—C12—H12A	120.8
N5—N4—N3	175.9 (7)		
			
N3—Zn1—O1—C1	−96.2 (5)	O1—C1—C6—C5	−178.0 (5)
N1—Zn1—O1—C1	83.7 (5)	C2—C1—C6—C5	0.6 (8)
N2—Zn1—O1—C1	0.8 (4)	O1—C1—C6—C7	2.6 (8)
O1^i^—Zn1—O1—C1	170.2 (5)	C2—C1—C6—C7	−178.8 (5)
N3—Zn1—O1—Zn1^i^	93.6 (2)	N2—C7—C6—C5	−174.0 (6)
N1—Zn1—O1—Zn1^i^	−86.5 (2)	N2—C7—C6—C1	5.4 (10)
N2—Zn1—O1—Zn1^i^	−169.34 (19)	C1—C6—C5—C4	0.4 (9)
O1^i^—Zn1—O1—Zn1^i^	0.0	C7—C6—C5—C4	179.8 (6)
N3—Zn1—N2—C7	120.0 (5)	C14—N1—C10—C11	1.8 (9)
O1—Zn1—N2—C7	6.0 (5)	Zn1—N1—C10—C11	174.3 (5)
N1—Zn1—N2—C7	−113.8 (5)	C14—N1—C10—C9	−178.9 (5)
O1^i^—Zn1—N2—C7	−36.6 (9)	Zn1—N1—C10—C9	−6.4 (7)
N3—Zn1—N2—C8	−70.6 (4)	O1—Zn1—N3—N4	−120.4 (7)
O1—Zn1—N2—C8	175.4 (4)	N1—Zn1—N3—N4	59.7 (8)
N1—Zn1—N2—C8	55.6 (4)	N2—Zn1—N3—N4	146.6 (7)
O1^i^—Zn1—N2—C8	132.9 (6)	O1^i^—Zn1—N3—N4	−39.5 (7)
N3—Zn1—N1—C14	−137.7 (5)	C7—N2—C8—C9	153.0 (6)
O1—Zn1—N1—C14	42.4 (5)	Zn1—N2—C8—C9	−16.9 (7)
N2—Zn1—N1—C14	129.0 (5)	C1—C2—C3—C4	−0.3 (10)
O1^i^—Zn1—N1—C14	−35.9 (5)	N1—C10—C11—C12	−1.3 (11)
N3—Zn1—N1—C10	49.9 (5)	C9—C10—C11—C12	179.5 (7)
O1—Zn1—N1—C10	−129.9 (4)	C10—N1—C14—C13	−1.1 (9)
N2—Zn1—N1—C10	−43.3 (4)	Zn1—N1—C14—C13	−173.3 (5)
O1^i^—Zn1—N1—C10	151.8 (4)	N1—C10—C9—C8	70.0 (7)
Zn1—O1—C1—C2	176.8 (4)	C11—C10—C9—C8	−110.8 (7)
Zn1^i^—O1—C1—C2	−15.3 (7)	N2—C8—C9—C10	−53.1 (7)
Zn1—O1—C1—C6	−4.6 (7)	C6—C5—C4—C3	−1.3 (11)
Zn1^i^—O1—C1—C6	163.2 (4)	C2—C3—C4—C5	1.2 (10)
C3—C2—C1—O1	178.0 (5)	N1—C14—C13—C12	−0.2 (11)
C3—C2—C1—C6	−0.6 (8)	C14—C13—C12—C11	0.7 (12)
C8—N2—C7—C6	−179.0 (5)	C10—C11—C12—C13	0.0 (12)
Zn1—N2—C7—C6	−9.7 (9)		

Symmetry codes: (i) −*x*+2, −*y*, −*z*.

### Hydrogen-bond geometry (Å, °)

**Table:**

*D*—H···*A*	*D*—H	H···*A*	*D*···*A*	*D*—H···*A*
C9—H9A···N5^ii^	0.97	2.58	3.198 (9)	122

Symmetry codes: (ii) −*x*+1, −*y*, −*z*.
